# Evaluation of the maternal deaths surveillance and response system at the health district level in Guinea in 2017 through digital communication tools

**DOI:** 10.1186/s12978-019-0671-3

**Published:** 2019-01-18

**Authors:** Tamba Mina Millimouno, Sidikiba Sidibé, Alexandre Delamou, Kéfilath Olatoyossi Akankè Bello, Basile Keugoung, Jean Paul Dossou, Abdoul Habib Beavogui, Bruno Meessen

**Affiliations:** 1 Centre National de Formation et de Recherche en Santé Rurale de Maferinyah, Forécariah, Guinea; 2 Health Services Delivery Community of Practice, Antwerp, Belgium; 3 Department of Public Health, Gamal Abdel Nasser University, Conakry, Guinea; 4 Centre de Recherche en Reproduction Humaine et en Démographie, Cotonou, Benin; 5 Institute of Tropical Medicine, Antwerp, Belgium

**Keywords:** Evaluation, Maternal death, Surveillance and response, Digital tools, Guinea

## Abstract

**Background:**

Reducing maternal mortality still remains a major challenge in low-income countries. This study aims to explore how digital communication tools can be used to evaluate the maternal deaths surveillance and response (MDSR) system at the health district level in Guinea.

**Methods:**

A descriptive cross-sectional study was conducted, using an innovative digital approach called District.Team, from April to September 2017. This study targeted all 38 district medical officers in Guinea. In addition to district medical officers, the participation of health actors from regional and central levels were also expected in the online discussion forum. Data collected through the questionnaire were mixed and those from the online discussion forum were entirely qualitative.

**Results:**

In total, 23 (61%) district medical officers (DMOs) participated in the study. Out of health districts (87%) which had updated guidelines and standards for the MDSR, 4 (20%) did not apply the content. In two health districts (8.7%), not all health facilities had maternal deaths notification forms. Three districts (13%) did not have maternal death review committees. In 2016, only half (50.2%) of reported maternal deaths were reviewed. The main recommendation formulated was related to quality of care. Other needs were also highlighted including continuous training of health care providers on emergency obstetric and neonatal care. Less than half (45%) of the review committee’s recommendations were implemented. Six health districts (26.1%) did not have a response plan to reported maternal deaths and no district annual report on the MDSR was published in 2016. The weaknesses identified were, among others, insufficiency of human resources and lack of financial resources. Fifty-eight messages related to MDSR weaknesses and improvement solutions were posted in the online discussion forum by 28 participants (23 DMOs and 5 health actors from regional and central levels).

**Conclusion:**

Digital tools can be used to assess the functioning of a system like maternal deaths surveillance and response. Moreover, the findings of the evaluation conducted will help stakeholders (starting from the health districts themselves) to design strategies and interventions for an effective MDSR.

## Plain English summary

Maternal deaths surveillance and response (MDSR) is a system of continuous surveillance that links the health information system and quality improvement processes from local to national levels. It includes the routine identification, notification, quantification and determination of causes and avoidability of all maternal deaths, as well as the use of this information to respond through actions that will prevent future deaths. In this study, an innovative digital approach (District.Team) was used to assess the organization and functioning of this MDSR system at the local (health district) level in Guinea.

After analyzing data collected through the questionnaire, results were published on the District.Team platform and then, respondents were invited to share their proposals of improvement solutions regarding to MDSR weaknesses, through an online discussion forum.

Of the 38 district medical officers, 23 (61%) participated in the study. Additionally, five health actors from regional and central levels responded in the online discussion forum.

Major challenges were highlighted including: poor implementation of MDSR guidelines, inadequate human and financial resources, undocumented maternal mortality ratio, deaths under-reporting, non-review of all maternal deaths, weak implementation of recommendations from the review committees and lack of response actions. Fifty-eight messages related to MDSR improvement solutions were posted in the online discussion forum.

In conclusion, digital tools can be used to assess the functioning of a system like maternal deaths surveillance and response. Stakeholders will be guided by the findings of this study in designing strategies and interventions for an effective MDSR system which can significantly reduce maternal mortality.

## Background

Approximately 830 women die every day in the world due to complications related to pregnancy or childbirth. Almost all of these maternal deaths (99%) occur in developing countries, and half in sub-Saharan Africa [1].

The maternal mortality ratio in developing countries was estimated at 239 [229; 275] per 100,000 live births in 2015, compared to 12 [11; 14] per 100,000 in developed countries [2]. The first target of Sustainable Development Goal 3 is to reduce the global maternal mortality ratio to below 70 per 100,000 live births by 2030 and no country should have a maternal mortality ratio greater than twice the world average of 140 deaths per 100,000 live births [1].

In Guinea, although significant progress has been made in reducing maternal mortality, it is still high, with a ratio of 550 deaths per 100,000 live births in 2016 [3]. One of the major challenges of the National Strategic xPlan for Reproductive, Maternal, Neonatal, Infant and Adolescent Health for the 2016–2020 period is the strengthening of high-impact interventions, particularly the Maternal Deaths Surveillance and Response (MDSR) [4].

MDSR encompasses continuous surveillance that links the health information system and quality improvement processes from the local to national levels. It includes the routine identification, notification, quantification and determination of causes and avoidability of a maternal death, as well as the use of this information to respond through actions that will prevent future deaths [5]. Therefore, MDSR requires a coordinated approach, ensuring that both the national- and district-level stakeholders are capacitated and supported to implement MDSR in a “no name, no blame” environment favorable to learning [6, 7].

More than 1 year after the official launch of the MDSR system in Guinea in 2016 [8], it was thus necessary to assess it in order to ensure that the major steps in the system are functioning adequately and improving with time [5]. In order to effectively involve field actors from the health districts and as part of a quick learning process, we decided to build on an innovative online approach called “District.Team”, set up since 2016, to promote horizontal learning between health district management teams (HDMTs) [9].

The purpose of this study was to explore how digital communication tools can be used to evaluate an intervention implemented at the health district level. In this case, we specifically planned to evaluate the MDSR system at the health district level in Guinea.

## Methods

A conceptual framework was adapted based on the World Health Organization (WHO) criteria for exploring the organization and implementation of the MDSR at the health district level [5]. These criteria are organized into four cyclic stages of the MDSR: i) Identification and Notification; ii) Review; iii) Analysis and Interpretation and iv) Response and Follow-up (Table 1). We then used the District.Team approach for the study design, data collection and analysis followed by an online discussion forum.

**Table 1:** Conceptual framework for the assessment of the maternal deaths surveillance and response, Guinea, 2017

Theme	Questionnaire	Online discussion forum
Guidelines and focal point	Existence and use of guidelines Existence of focal point	Weaknesses Solutions
Identification and notification of maternal deaths	Notification forms Community notification Notification of maternal deaths within 24 h Means for notification (paper-based, SMS, phone, email) Number of maternal deaths reported in the districts (and within 24 h)	
Review of maternal deaths	Review committee (Existence and composition) Implementation of the review	
Analysis and Interpretation	Data analysis and formulation of recommendations Maternal mortality ratio	
Response and follow-up	Implementation of recommendations Response plan Publication of annual report	

### Overview of the “District.Team approach”

District.Team was part of a big action-research project called “Mobilization 2.0” which was developed by the Health Services Delivery Community of Practice (HSD CoP) and implemented in Guinea and Benin from January 2016 to September 2017 [10].

**Fig. 1:**
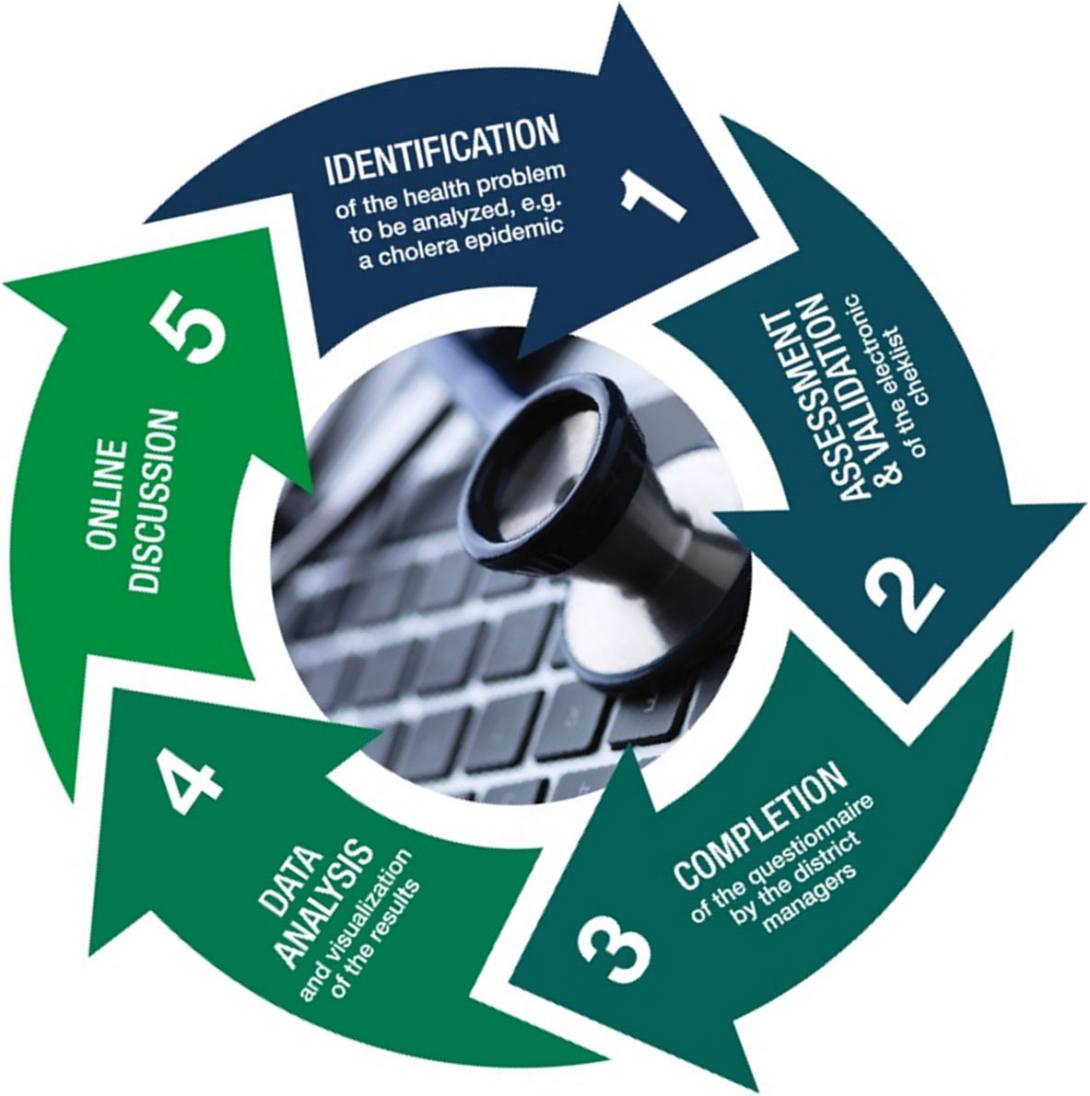
Steps of a collective learning process on District.Team

The modus operandi and effectiveness of the District.Team approach have been extensively reported in another paper (Keugoung et al) [9]. The overall goal of this approach is to enhance cross-learning between HDMTs in order to improve the response to epidemic-prone diseases and other health challenges at the decentralized level. Through District.Team, we implement an open interaction between HDMTs using digital tools such as email, SMS, online discussion forum and the electronic platform District.Team. The collective learning is organized as a cyclic iterative approach. Each cycle has five steps: i) Identification of a health issue to investigate; ii) Elaboration of the online questionnaire by the facilitation team using the Google form tool; iii) Administration of the questionnaire; iv) Data analysis, production and publication of results as visualizations at the online platform (http://guinee.district.team/ for Guinea); and finally v) Online discussion forum on results (Fig. 1). In this article, we report the outcome of one of the most recent cycle on District.Team/Guinée: the assessment of the Guinean MDSR.

### Study design

We conducted a descriptive cross-sectional study. The electronic questionnaire was developed using the conceptual framework and completed between April and September 2017. After publishing the results as a blog on the District.Team/Guinée platform, we organized an online discussion forum with the participants in the study from February, 7 to March, 112,018. This online discussion forum was about the key questions that emerged related to the weaknesses of the MDSR system in order to have proposals for improvement solutions.

### Setting

Guinea is located in West Africa and had a population of 10,523,261 inhabitants in 2014 [10]. The Guinean health system has three levels: local (district), intermediate (region) and central (Ministry of health). There are 8 regions and 38 health districts [11]. The health districts’ population varied from 102,866 (Fria) to 918,043 (Matoto) inhabitants in 2016. Each health district is administered by a health district management team (HDMT) whose role includes planning, supervising, monitoring and evaluating district health activities [11]. A health district management team is headed by a District Medical Officer (DMO), who is called the Prefectural Director of Health in the Prefectures and the Communal Director of Health in Conakry. In 2016, coverage of antenatal care carried out by trained staff was 84% and that of assisted childbirths by trained staff was 63% [3].

### Study population and sampling

An informational email was sent to all 38 DMOs by the District.Team/Guinée national coordinator (TMM). The DMOs who provided a written informed consent for participation were included in the study.

### Data collection methods

An electronic questionnaire (self-administered) and an online discussion forum were used to collect data. The language of administration was French. The questionnaire (Google form) made of open-ended and multiple choice questions focused on the four cyclic stages of the MDSR, was sent through emails to district medical officers. An active follow-up of the questionnaire filling was made by the Guinean research team (TMM, SS, AD) using phone short messages service (SMS), emails and phone calls according to the District.Team strategy. After sending a first email (on Tuesday), an SMS was sent 24 h later (on Wednesday), a second email was sent 48 h later (on Thursday) and a phone call carried out 78 h later (on Friday). This process was repeated weekly for three consecutive weeks during the first month of data collection and then every first week of the last 5 months. Additionally, an online discussion forum was organized on the District.Team/Guinée platform where all participants (DMOs, some staffs of the regional and central levels) were already registered. Participants shared their views on the weaknesses of the MDSR system and improvement solutions.

### Analysis

Data collected through the questionnaire were centralized in Google Sheets, then downloaded in Microsoft Excel 2016 format and cleaned. Statistical analyses were performed using Epi Info version 7 of the CDC of Atlanta. Proportions without confidence interval were used because of small sample size to summarize the variables. These data were analyzed based on the conceptual framework (Table 1). Data from the online discussion forum were summarized, organized by theme and integrating also some quotes from participants.

### Ethical considerations

The research protocol was approved by the National Ethic Committee for Health Research in Guinea (N°: 49/CNERS/16). The objectives of the research were explained to all DMOs prior to its start and gave their consent at the beginning of the questionnaire in a section designed for this purpose.

## Results

**Fig. 2:**
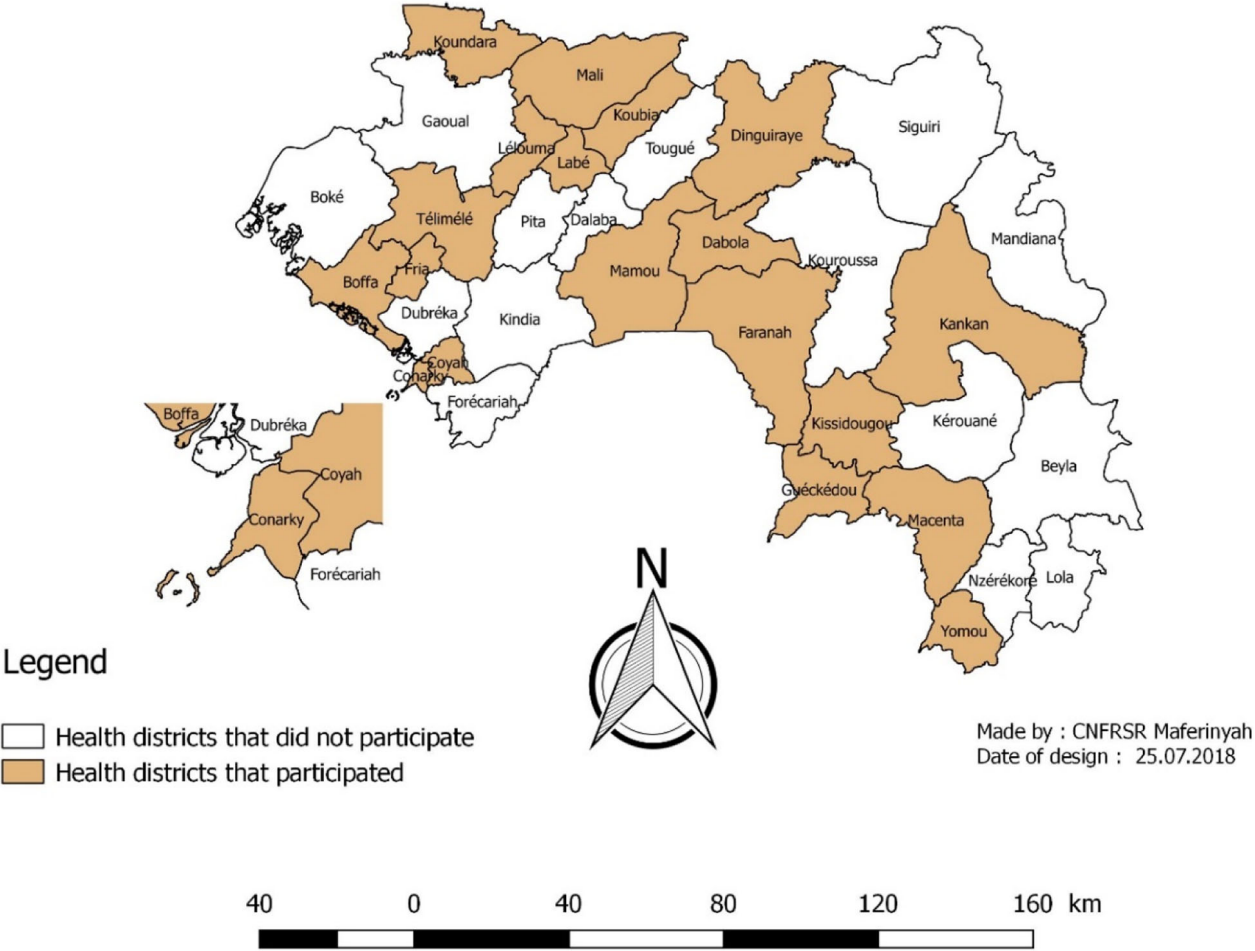
Participation of health districts in the study on Maternal Deaths Surveillance and Response in Guinea, 2017

Out of the 38 DMOs contacted via e-mail, 23 responded and participated in this study, representing a response rate of 61% (Fig. 2). During the follow up of the questionnaire filling, almost half (30%) of the respondents were mobilized by e-mails, 10% by phone short messages and 21% by phone calls. In the online discussion forum, 28 respondents (23 DMOs and 5 health actors from regional and central levels) posted 58 messages related to the understanding of the MDSR weaknesses in Guinea and improvement solutions.

Through these digital tools and following the conceptual framework, the organization and implementation of the MDSR were evaluated which results are summarized as follows:

### Guidelines and focal point

The majority (87.0%) of responding districts had updated guidelines and standards of MDSR. On the other hand, three districts (13%) did not have this reference document. Most (80%) districts with updated MDSR guidelines and standards applied them. The four districts (20%) that did not apply these guidelines and standards did not mention any reason for non-application. However, through online discussion forum the participants gave the following reasons for not using the guidelines: insufficient training of staff on updated guidelines and standards, lack of supervision, lack of accountability spirit, negligence of managers, lack of professionalism of actors and leadership, misinformation of health staff and the community about MDSR, lack of dynamism and rigor in the work, fear of blame and punishment resulting in habitual under-reporting.

All districts (100%) had reproductive health focal points: solely at district office level (39.1%); at the district office and at each health facility (17.4%); at the district office, at each health facility and in each community (39.1%); solely in each health facility (4.4%). No district had a specific MDSR focal point neither at the district office nor at facilities level (Table 2).

**Table 2:** Organization of MDSR in health districts in Guinea, 2017 (*N* = 23)

Variables	Number	Percentage (%)
Focal point		
Existence of the Reproductive Health focal point at the district level		
Health district office solely	9	39.1
Health district office and each health facility	4	17.4
Health district office, each health facility and each community	9	39.1
Each health facility solely	1	4.4
No focal point	0	0
Existence of the specific MDSR focal point (district office and facilities level)		
Yes	0	0
No	23	100.0
Maternal deaths notification		
Existence of maternal deaths notification forms		
Yes	21	91.3
No	2	8.7
Community-based maternal deaths surveillance		
Yes	21	91.3
No	2	8.7
Notification of maternal deaths made mandatory within 24 h		
Yes	21	91.3
No	2	8.7
Means of maternal deaths notification at the district level		
Phone calls	4	17.4
Phone calls and phone messages (sms)	4	17.4
Phone calls and paper declaration	7	30.4
Phone calls, sms and paper declaration	8	34.8
Maternal deaths review		
Existence of review committee at the health district level and facilities		
Yes	20	87.0
No	3	13.0
Multidisciplinary composition of the review committee (*n* = 20)		
Medical doctors, nurses/midwives and communities	13	65.0
Medical doctors, nurses/midwives	3	15.0
Medical doctors, nurses/midwives, communities and family	4	20.0
Review (audit) of all notified maternal deaths at the first trimester (*n* = 20)		
Yes	15	75.0
No	5	25.0
Reasons for not reviewing all maternal deaths (*n* = 5)		
Insufficiency of financial ressources	5	100.0
Insufficiency of human resources	5	100.0
Interference of activities (Overload of work)	2	40.0
If the deceased comes from another heath district, often parents are in a hurry	1	20.0
Types of data bases of maternal deaths		
Electronic register	12	52.2
Paper register	3	13.0
Electronic and paper register	8	34.8
Response plan		
Existence of response plan		
Yes	17	73.9
No	6	26.1
Reasons for non-existence of a response plan (*n* = 6)		
Lack of financial support to the health district for maternal deaths reviews	4	66.7
Insufficiency of human ressources	2	33.3

### Identification and notification of maternal deaths

In two districts (8.7%), not all health facilities had notification forms of maternal deaths. Community-based maternal deaths surveillance was organized in 91.3% of health districts. However, it was not done in 8.7% of districts. In health facilities of the majority (91.3%) of districts, notification of maternal deaths was made within 24 h. Only in health facilities of two districts, this was not the case. For the notification of maternal deaths, in 34.8% of districts, health facilities and communities used various means including phone calls, SMS and the paper declaration; in 30.4% of districts health facilities used either the phone calls or the paper declaration; in 17.4% of districts health facilities did so by phone call or SMS and in 17.4% of districts health facilities used only the phone call (Table 2).

In 2016 (Table 3), 265 maternal deaths were reported in 21 health districts where notification was in place in health facilities and communities. Among these deaths, 230 (86.8%) were notified within 24 h.

**Table 3:** Implementation of MDSR in health districts in Guinea, 2016 (*N* = 23)

Variables	Number	Percentage (%)
Notification and review of maternal deaths		
Notification of maternal deaths in health facilities and communities		
Maternal deaths notified	265	100.0
Maternal deaths notified within 24 h	230	86.8
Review of maternal deaths in health facilities and communities (*n* = 265)		
Maternal deaths reviewed	133	50.2
Maternal deaths unreviewed	132	49.8
Recommendations		
Implementation of the recommendations of the district review committee		
Yes	9	45.0
No	11	55.0
Implementation of the recommendations of the review committees related to the review of notified maternal deaths		
Yes	3	15.0
No	17	85.0
Recommendations on which the health districts focused		
Training of members of the Maternal Deaths Review Committees	13	65.0
Financial support for MDSR activities carried out (Training, Review, Monitoring and Evaluation)	16	80.0
Reference of all complicated cases on time	10	50.0
Improvement of reference and counter-reference	9	45.0
Motivation of village birth attendants and community relays for referral of women to health facilities	12	60.0
Engagement of the community in mutual health	5	25.0
Facilitation of the transportation of pregnant women through collaboration with the union of carriers	9	45.0
Continuous training of health care providers in emergency obstetric and neonatal care	14	70.0
Provision of health facilities in equipment and medicines	12	60.0
Promptness in reporting health data	13	65.0
Dissemination of the recommendations of the different reviews	11	55.0
Maternal mortality ratio		
Calculation of maternal mortality ratio of the district		
Yes	4	17.4
No	19	82.6
MDSR report		
Publication of the annual report on the MDSR		
Yes	0	0.0
No	23	100.0
MDSR weaknesses		
Weaknesses in implementing or functioning the MDSR system		
Insufficiency of human resources (in number and quality)	8	34.8
Insufficiency/Lack of financial resources	6	26.1
Interference of activities	1	4.3
Low meeting rate on maternal deaths (once per semester)	1	4.3
Lack of information on the functioning of the MDSR system	4	17.4
Insufficiency of dissemination of guidelines and standards in MDSR (updated)	3	13.0

### Review of maternal deaths

The majority (87%) of health districts had maternal deaths review committees at health facilities and at the district level. These review committees are multidisciplinary in general and include medical doctors, nurses, midwives, communities and families. Three districts (13%) did not have a maternal death review committee either at health facilities or at the district level. In most districts (75%) with review committees at health facilities and at the district level, all reported maternal deaths were audited in the first trimester of 2017 while in 25% of the districts only some maternal deaths were audited. The main reason for not reviewing all maternal death cases was the insufficient financial and human resources. All responding districts had databases of maternal deaths of different types in health facilities and at the district office: more than half (52.2%) of districts used only an electronic registry; More than one-third (34.8%) of districts had both an electronic and paper registry and only 3 districts (13%) used only a paper registry (Table 2).

In 2016, among the maternal deaths notified, only half (50.2%) was reviewed (Table 3).

### Analysis and interpretation

The majority (82.6%) of districts did not calculate the maternal mortality ratio in their districts in 2016. In all districts, there were recommendations related to quality of care. Some districts also highlighted other needs such as funding of MDSR activities (training, review, monitoring and evaluation) (80%), continuous training of health care providers on emergency obstetric and neonatal care (70%), training of the members of the maternal deaths review committee (65%), promptness in reporting health data (65%), motivation of village birth attendants and community relays for referral of women to health facilities (60%), provision of equipment and medicines to health facilities (60%), dissemination of the recommendations of the different reviews (55%), Reference of all complicated cases on time (50%), facilitation of the transportation of pregnant women through collaboration with the union of carriers (45%), improvement of reference and counter-reference (45%) and engagement of the community in mutual health (25%) (Table 3).

### Response and follow-up

In 2016, on average, less than half (45%) of recommendations of review committees were actually implemented at the district level. Only 15% of the districts implemented all recommendations of the review of maternal deaths (Table 3). In 17 districts (73.9%), there was a response plan developed in response to reported maternal deaths, while 6 districts (26.1%) did not have one. The main reasons given for this lack of a response plan were the lack of financial support to the districts for the review of maternal deaths (66.7%), followed by insufficient human resources (33.3%) (Table 2). No district committee published an annual report on the maternal deaths surveillance and response in 2016 (Table 3).

### Weaknesses in implementing MDSR

Weaknesses in fully implementing the MDSR system identified by the districts were – insufficiency of human resources (34.8%), insufficiency/lack of financial resources (26.1%), lack of information on the functioning of the MDSR system (17.4%), insufficiency of dissemination of updated guidelines and standards of MDSR (13%), low meeting rate on maternal deaths at the district level (once per semester, during the meeting of the health district technical committee) (4.3%) and interference of activities (4.3%) (Table 3).

### Solutions to improve the MDSR system

Participants in the online discussion forum proposed solutions to improve the MDSR in Guinea including the followings:

Firstly, in relation to the insufficient dissemination of documents (updated guidelines and standards in MDSR), it was suggested that this activity be decentralized to the regional and district levels under the supervision of the national directorate of family health and nutrition and partners (technical and financial). A regional director of health said “*in my opinion, from now as all the health districts have been equipped with computer equipment, the electronic versions of the documents should just be sent to DMOs. They can make copies and share them to their own staffs”.*

Secondly, regarding to the non-application of the guidelines and standards in MDSR, they proposed to – strengthen the joint formative supervisions (regional directorates of health and partners); recommend strongly the application of documents’ content; introduce rigorously MDSR into routine activities through registers; set up a monitoring and evaluation framework at the central, regional and district levels; involve strongly the community.

Afterwards, concerning the underreporting and the weak review of maternal deaths, participants suggested to make health workers understand that the main purpose of the review is not to punish but to improve the quality of care, provide the best care to patients and establish good collaboration. “*In most cases, providers do not complete the death notification forms. I take this opportunity to remind the heads of health facilities and district medical officers to make a communication on the review of maternal deaths in order to have the real determinants of maternal deaths*” declared a health actor from Ministry of Health. Improvement tracks related to the non-review of all maternal deaths reported are – to empower a member of the HDMT to follow up on the responsibilities of the review committee; to make requests to stakeholders for financing review activities; and to better plan activities in the district.

Furthermore, the non-implementation of the recommendations made by the review committees is another crucial challenge as highlighted as well by A DMO: “*Thank you very much, colleagues, for the hard work and courage especially because maternal deaths remain a weak link in our health system even though the World Health Organization says the woman must not lose her life giving life. Our district in 2017 registered 19 maternal deaths, of which 13 were reviewed but how many recommendations were followed??”.* To address this issue, participants suggested to the health authorities to involve in supervision. “*The national level needs to be involved in supervision through its “Safe Motherhood” programme responsible for monitoring and evaluating the MDSR. It is important that each health actor plays his part if we want to achieve real change in our health system*” as affirmed by another health actor from Ministry of Health.

In addition, to deal with lack of maternal deaths response plan, participants proposed to integrate maternal deaths response plan into operational action plan, search for funding through a request addressed to stakeholders and proceed to its implementation.

Finally, for an effective MDSR system, participants suggest that focal points of MDSR should be appointed at district level, in each health region and at the Ministry of Health with respective responsibilities and an appropriate training. Overall, these focal points should ensure the implementation of the MDSR including the training and supervision of health workers on the guidelines and standards, surveillance of zero notification, quality control of maternal deaths notification, execution of the review of each case of maternal death, dissemination of the recommendations of each review, follow-up of the implementation of recommendations made by the review committees, dissemination of the MDSR report of the district by trimester/semester/year, etc. And the District.Team/Guinée platform could be used to disseminate the review reports and create debate (discussion forums) for an exchange of experiences between health districts.

## Discussion

This study highlighted major challenges in the MDSR system in Guinea, which still abide. These include: poor implementation of guidelines, inadequate human resources, undocumented maternal mortality ratio, non-review of all maternal deaths, weak implementation of recommendations from the review committees and lack of response actions.

It underpins the need for close monitoring of health district activities to improve their performance. Some of the factors of poor implementation of guidelines such as misinformation can be easily addressed through the online discussion forum.

The under-reporting of maternal deaths due to providers’ fear of being blamed and punished as also observed in Malawi [12, 13], can be solved by a clear understanding of the MDSR objectives.

As MDSR is included in the integrated disease surveillance and response (IDSR), the person in charge of the epidemiological surveillance is the one in charge of the maternal deaths notification to avoid duplication in a context of scarce human resources [5, 6]. But, there is no focal point neither at the district office nor in health facilities, who monitors the implementation of reviews and resulting recommendations. However, it is important to assign MDSR responsibility to a specific member of the HDMT who should ensure that the four stages of MDSR are properly implemented in the entire health district. In Malawi, the appointment of district MDSR leaders has improved the response to the maternal death review. For instance, in 2014 in the district of Mchinji, 67% of the recommendations were followed, compared to 26% in 2013 [14].

Only one out of five health districts calculated their maternal mortality ratio in 2016. Though, identifying all maternal deaths in a given area is imperative for having a real maternal mortality ratio and assessing the effectiveness of the MDSR [5].

Three out of four heath districts that had a review committee audited all maternal deaths reported in the first quarter of 2017. This is certainly an improvement compared to 2016 in Guinea and to 2014 in Kenya [15], when only half of maternal deaths notified were audited respectively 50.2% et 51.2%. We could explain that improvement by the strengthening of health district capacities in terms of recruiting and deploying health workers early 2017.

As reported in several studies [16–19], the main reason for not reviewing all reported maternal deaths was the insufficiency of financial and human resources (100%).

In Guinea, there is no maternal deaths review committee at the community level. Creating maternal deaths review committees at the community level could increase the number of maternal deaths reviewed. In Malawi, a pilot programme conducted in 2011–2012 in the Mchinji health district showed that a community-based approach doubled the number of maternal deaths audited [12]. In 2012, another community-based approach for maternal deaths review reported that three out of four maternal deaths recorded were reviewed in India [20].

No district committee published an annual report on the MDSR in 2016. While, annual national and district reports that summarize MDSR results, recommendations, and the actions taken are a critical component of MDSR [5, 21]. An annual report is also a response in and of itself, because it feeds into the planning process and can contribute to health system reforms or to the design of new and innovative interventions [5].

This study also confirmed the feasibility of using digital tools to rapidly evaluate the implementation of an intervention at the district level, report the obstacles and organize a collegial discussion in order to generate the possible solutions for a better implementation as the case of MDSR.

Through the District.Team approach, we used low-cost digital means (email, phone short messages, phone calls, online discussion forum and the District.Team platform) to mobilize DMOs and some health actors from regional and central levels around the MDSR for its evaluation. This highlighted a better understanding of the MDSR weaknesses and improvement tracks. Hence, the District.Team approach used to conduct this evaluation were beneficial in terms of resources (human, material and financial). This approach could also be beneficial in time, when District.Team finds an institutional anchor and integrates existing health programmes, because this will stimulate and regularize the participation of DMOs/HDMTs and reduce data collection time [9].

In Guinea, an option for the future related to institutional anchoring of District.Team could be through the Office of Strategy and Development of the Ministry of health. Collaboration between District.Team and DHIS2 platforms is another strategy for facilitating data collection and use for decision-making.

Ensuring government accountability for improving maternal health requires the periodic and transparent dissemination and discussion of key results, particularly trends in maternal mortality [5].

Thus, District.Team provides a favorable environment for developing a learning health system. Indeed, it incorporates some elements of the three steps – capacity to collect new information; generation of a new understanding and synthesis; and lastly adaptation of action- needed to build a learning organization [22].

### Limitations of the study

The study has some limitations. Data is based on information provided by DMOs through a self-administered questionnaire. Information on the MDSR was not directly observed; therefore, they could have been over- or underestimated by DMOs or otherwise biased. The status of the MDSR at the central level (Ministry of Health) and this of incorporating perinatal or neonatal deaths in this system have not been explored, although additional lessons should be learned. Our sample is not necessarily representative, this should affect the values of the confidence intervals.

## Conclusion

District.Team approach has been used for a rapid assessment of MDSR in Guinea at the health district level. It could also be used for further iterative assessments of MDSR as well as other health issues at the district level in resource limited settings. This study provided better knowledge in maternal deaths surveillance and response at the operational level in Guinea. Challenges persist at all stages of the MDSR system. Recommendations or solutions reflecting the realities of the health districts have been proposed to overcome these challenges, and should be used to update the national action plan of MDSR. Effective use of the lessons learned from this study for decision-making at all levels of the health system is a cornerstone that contributes to the elimination of avoidable maternal mortality in health facilities and communities. Further studies are needed to explore the coordination of MDSR at the central level and the incorporation of perinatal or neonatal deaths into this MDSR system for a better understanding of its practical functioning.

## Abbreviations

DHIS2: District Health Information Software, version 2
DMO: District medical officer
HDMT: Health district management team
HSD CoP: Health Services Delivery Community of Practice
IDSR: Integrated disease surveillance and response
MDSR: Maternal death surveillance and response
SMS: Short message service
